# Cognitive impairment associated with MAPK inhibition in colorectal cancer

**DOI:** 10.1192/j.eurpsy.2026.11544

**Published:** 2026-07-31

**Authors:** E. Gevorkyan, A. Bobrov, N. Semenova, L. Vladimirova

**Affiliations:** 1 V. Serbsky National Medical Research Centre for Psychiatry and Narcology, Moscow; 2 National Medical Research Centre for Oncology, Rostov-on-Don, Russian Federation

## Abstract

**Introduction:**

Targeted therapy significantly improves survival in metastatic colorectal cancer (mCRC). However, its neuropsychiatric side effects remain poorly understood. The mitogen-activated protein kinase (MAPK) cascade plays a crucial role in synaptic plasticity and memory formation; therefore, its inhibition through targeted therapy, including EGFR blockade, is a matter of interest for studying patients with mCRC.

**Objectives:**

This study aimed to evaluate the impact of anti-EGFR-targeted therapy, compared with anti-VEGF-targeted therapy, on cognitive performance in patients with stage IV colorectal cancer.

**Methods:**

Eighty patients with mCRC were enrolled. The main group (n = 55) received FOLFOX6 combined with an anti-EGFR monoclonal antibody (cetuximab or panitumumab), which inhibits the MAPK cascade. The control group (n = 25) received FOLFOX6 plus bevacizumab, which does not affect the MAPK cascade. Cognitive status was assessed at baseline and after 3–4 months using the Addenbrooke’s Cognitive Examination-Revised (ACE-R) and the Frontal Assessment Battery (FAB).

**Results:**

At baseline, cognitive scores were comparable between groups. After 3–4 months, the anti-EGFR targeted group showed a marked decline in ACE-R total scores from 94[90; 95] to 86[85; 90] (p = 0.001), whereas the control group remained stable (94[91; 97] to 94[90; 96]; p> 0.05). Memory scores declined in the anti-EGFR group from 22[22; 24] to 19[19; 20] (p= 0.001), while the control group showed only a mild reduction (23[21; 26] to 22[21; 25]; p= 0.034). Attention/orientation also decreased in the anti-EGFR group (18[16; 18] to 17[15; 18]; p= 0.024), but remained unchanged in controls (18[18; 18] throughout). Other ACE-R domains and FAB subscores demonstrated no significant changes.

**Conclusions:**

Anti-EGFR-targeted therapy, which involves inhibiting the MAPK cascade, is found to be associated with selective cognitive impairment, predominantly affecting memory and attention. Regular cognitive monitoring may support timely psychiatric referral, optimize management strategies, and ultimately improve patients’ quality of life.

**Disclosure of Interest:**

None Declared

